# Comprehensive analysis and expression characterization of potato Cytochrome P450 gene family and the role of *StCYP67* in abiotic stresses

**DOI:** 10.1186/s12870-025-07392-y

**Published:** 2025-09-20

**Authors:** Xiangyan Zhou, Caijuan Li, Rong Miao, Yanming Ma, Dan Zhang

**Affiliations:** 1 College of Life Science and Technology, Gansu Agricultural University, Lanzhou, 730070 China; 2 Gansu Provincial Key Laboratory of Aridland Crop Science, Gansu Agricultural University, Lanzhou, 730070 China

**Keywords:** Potato, Cytochrome P450, Bioinformatics, Abiotic stress, Gene expression

## Abstract

**Background:**

Brassinosteroids (BRs) play crucial roles in regulating developmental programming and activating stress-responsive networks under abiotic stress conditions. Members of cytochrome P450 monooxygenase (CYP450) superfamily have been reported to widely participate in the synthesis pathway of BRs in plants. However, the CYP450 superfamily needs to be systematically characterized at the whole-genome level in potatoes (*Solanum tuberosum*).

**Results:**

In this study, we identified 558 *StCYP450* genes using data from the DM v6.1 potato genome database. These genes were classified into nine clans encompassing 44 families, with *StCYP67* belonging to the CYP85 clan. Through bioinformatics analysis, we Mapped these genes across all 12 potato chromosomes and cell scaffolds. Phylogenetic reconstruction revealed that potato *StCYP450* genes clustered into nine evolutionary groups, with distinct enrichment in groups I and IX compared to other species. Promoter analysis identified multiple *cis*-acting regulatory elements linked to phytohormone signaling (e.g., ABA, BRs) and abiotic stress adaptation. Expression profiling demonstrated differential regulation of StCYP450 genes under Cd²⁺, 24-epibrassinolide (EBL), and ABA treatments. Notably, overexpression of *StCYP67* in potatoes significantly increased EBL content in transgenic potatoes (OE) compared with non-transgenic plants (NT), corroborated by RNA-seq data showing upregulation of BRs biosynthesis pathway genes. These findings strongly implicate StCYP450 proteins, particularly StCYP67, in mediating abiotic stress responses and BR synthesis in potatoes.

**Conclusion:**

558 members of the StCYP450 family in potato were identified, many of which appear to participate in abiotic stress responses. Functional validation of *StCYP67* highlights its dual role in BRs biosynthesis and stress regulation. These results not only clarify the evolutionary relationships within the StCYP450 gene family but also establish a foundation for future functional studies of these genes in potato.

**Supplementary Information:**

The online version contains supplementary material available at 10.1186/s12870-025-07392-y.

## Introduction

Cytochrome P450 (CYPs) superfamily, comprised of monooxygenase-encoding genes [1, 2], is currently the most extensive group of enzymes participating in plant metabolic processes [3]. Based on protein sequence similarity and phylogenetic analysis, CYP genes are divided into eleven clans, including seven single-family groups (CYP51, CYP710, CYP711, CYP74, CYP727, CYP746, and CYP97) as well as groups containing four multiple families (CYP71, CYP72, CYP85, and CYP86) [1, 4]. The CYP450 gene family contains a conserved heme-binding domain (FxxGxRxCxG) [5]. To date, over 20, 000 members of the CYP family have been classified into the CYP450 gene family, but their functions remain to be fully elucidated [6].

CYP proteins participate in numerous biosynthetic pathways of various compounds, such as alkaloids, flavonoids and antioxidants [7]– [8]. For example, the gene families CYP90, CYP724 and CYP734 contribute to synthesis of steroids and glycoalkaloids [9]. Additionally, CYP450s play a crucial role in the biosynthesis and degradation of plant hormones, such as brassinosteroids (BRs), and are essential in regulating plant responses to both biotic and abiotic stressors [10]. Specifically, C-3 oxidase (CYP90A) and C-22 hydroxylases (CYP90B1) are key rate-limiting enzymes in the BRs biosynthesis pathway [10], influencing BRs levels and regulating responses to salinity, drought, and biotic stresses [11]– [12]. For instance, overexpression of *StCYP90A* enhanced drought stress tolerance in transgenic potatoes [12], while downregulation o*f StCYP90B1* (referred to as *StCYP67* in this study based on its chromosomal distribution) via RNAi conferred salt tolerance in potatoes by enhancing antioxidant enzyme activities [13]. It should be noted that the naming principles of *CYP90A* and *CYP90B1* in the CYP450 superfamily are based on standardized evolutionary homology criteria established by Nelson et al. [14]. But the nomenclatures of *StCYP* genes in this study were according to their chromosomal locations.

Globally, potato (*Solanum tuberosum* L.) is recognized as a vital crop for both food production and industrial applications [15]. However, its productivity is severely limited by various abiotic and biotic stresses [16].

The CYP450 gene family has been extensively studied in diverse plants, such as *Citrus reticulata* [17], *Tartary buckwheat* [18], *Dendrobium huoshanense* [19], *Ipomoea batatas* [20], and *Brassica oleracea* [21]. However, the roles of StCYP450 genes in potatoes in responses to stress require further investigation.

Therefore, it is essential to investigate the gene structure of StCYP450 and its role in abiotic stress response. In this study, the structural features, conserved motifs, chromosome locations, evolutionary connections, *cis*-regulatory elements, and protein interaction networks of StCYP450 genes were systematically analyzed. Additionally, the expression profiles of StCYP450 genes in response to exogenous 24-epibrassinolide (EBL), abscisic acid (ABA), and cadmium (Cd) treatments were examined using online RNA-seq data and qRT-PCR. Their expression patterns under Cd^2+^ treatments were further explored at the transcriptomic level. Stable transformation of potato was employed to investigate the functions of *StCYP67*. The results provide valuable insights for further understanding the functional roles of StCYP450 genes.

## Results

### Identification of StCYP450 genes in potato

In this study, 558 StCYP450 genes were identified from the PF00067 domain (FxxGxRxCxG). The StCYP genes were designated as *StCYP1* to *StCYP558* based on their chromosomal locations (Table S1).

As shown in Table S1, StCYP proteins ranged from 69 (StCYP356) to 2548 (StCYP319) amino acids (AA) in length. Their molecular weights (MW) ranged from 7.856 to 289.360 kDa, respectively. The theoretical isoelectric points (pI) were between 4.47 (StCYP211) and 10.07 (StCYP109 and StCYP550). The grand averages of hydropathicity (GRAVY) values of StCYP proteins were mostly negative, indicating that they were predominantly hydrophilic proteins. The instability index (II) varied from 19.41 (StCYP501) to 69.66 (StCYP92), and the aliphatic index (AI) was between 62.47 (StCYP547) and 118.53 (StCYP291).

Subcellular localization predictions revealed that 274 (49.1%) StCYP proteins were located in the cytoplasm (CP). Additionally, 10.6% of the StCYP proteins were distributed in the nucleus or cytoplasm. Eleven were situated in the endoplasmic reticulum (ER), and only six and seven were located in the extracellular matrix (E) and vacuole (V), respectively. The remaining StCYP450 genes were found in mitochondria, cytoskeleton, plasma membrane, peroxisome and Golgi apparatus. These results suggested that StCYP450 proteins played a pivotal part in potatoes.

### Gene conserved motifs and structures of CYP450 proteins in potato

Gene structure domains and motifs of multi-gene families often diverge during evolution [22]. Generally, differences in protein motifs lead to variations in protein function [23–25]. To elucidate the phylogenetic relationships and structural patterns of the *StCYP450* genes, exon-intron and motif characteristics were analyzed by aligning coding sequences (CDSs) to genomic sequences using TBtools. In this study, highly conserved motifs were identified in StCYP proteins using MEME. Each *StCYP450* gene contained ten conserved motifs (motifs 1–10) (Fig.S1B), suggesting that these motifs may be essential for the function of the StCYP450 gene family. Over 80% of the StCYP450 genes contained six motifs. The close evolutionary relationships and reliable phylogenetic clustering of StCYP450 genes were further validated by the presence of highly conserved motifs and similar gene structures within the same family (Fig.S1A).

The exon-intron structure provides essential clues into the evolutionary features and functional divergence of genes [26]. The exon-intron organizations of *StCYP* genes were predicted (Fig.S1C). It was found that 85 *StCYP* genes lacked introns. Additionally, the number of exons in *StCYP* genes differed widely, ranging between 1 and 43. Analysis revealed that closely related *StCYP* genes tended to have similar exon structures. Specifically, 37.81% (211 of 558) of the *StCYP* genes contained three to seven exons, while 16.85% (94 of 558) of the *StCYP* genes contained only one exon. *StCYP319* had the largest number of exons (43). Some *StCYP* genes exhibited atypical gene structures compared to other members of the same family. For instance, *StCYP71A3*, *StCYP71B26*, and *StCYP79B1* in clan 71, as well as *StCYP72A17* in clan 72, had very short exons but very long introns. In brief, the exon-intron structures of *StCYP* genes varied significantly. Examples included *StCYP18*, *StCYP82*, *StCYP 286*, *StCYP384*, *StCYP399* in clan 71, *StCYP96*, *StCYP318* in clan 72, *StCYP355* in clan 85, *StCYP80* in clan 86.

### Chromosomal distribution analysis of StCYP450 genes in potato

Chromosome distribution analysis revealed that 553 StCYP450 genes were unevenly distributed across 12 chromosomes, while five genes (*StCYP554*-*558*) were located on scaffolds. Some genes were clustered on chromosomes 1–12, Likely due to tandem gene duplication. Chromosome 4 (Chr4) harbored the highest number of CYP450 genes (94), whereas chromosome 5 (Chr5) contained the fewest, with only 15 StCYP450 genes (Fig.S2).

### Synteny analysis of StCYP72 and StCYP85 gene clans

**Fig. 1:**
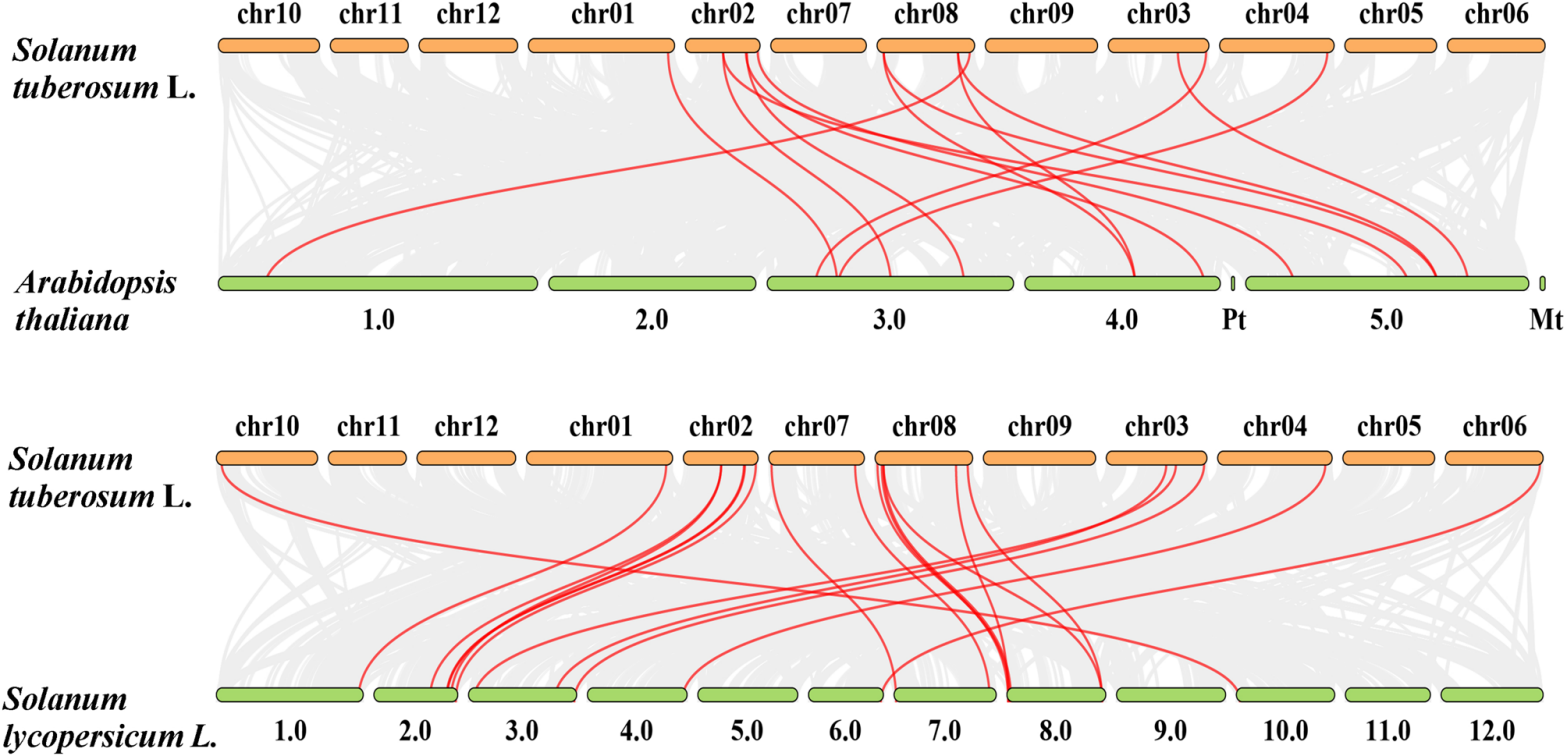
Synteny analysis of CYP72 and CYP85 clan genes among potato, Arabidopsis, and tomato. Gray lines in the background represent collinear blocks within each genome, while red lines highlight syntenic CYP450 gene pairs specifically between potato and Arabidopsis or potato and tomato

To investigate the syntenic relationships between CYP72 and CYP85 clan genes across potato, tomato (*Solanum lycopersicum* L.) and *Arabidopsis thaliana*, collinear genetic relationships were constructed using TBtools with MCScanX. This analysis revealed 14 collinear gene pairs between potato and Arabidopsis and 17 collinear gene pairs between potato and tomato (Fig. 1). These results indicated that potatoes and tomatoes shared a closer genetic relationship in terms of evolution.

### Phylogenetic examination of CYP450 genes

**Fig. 2:**
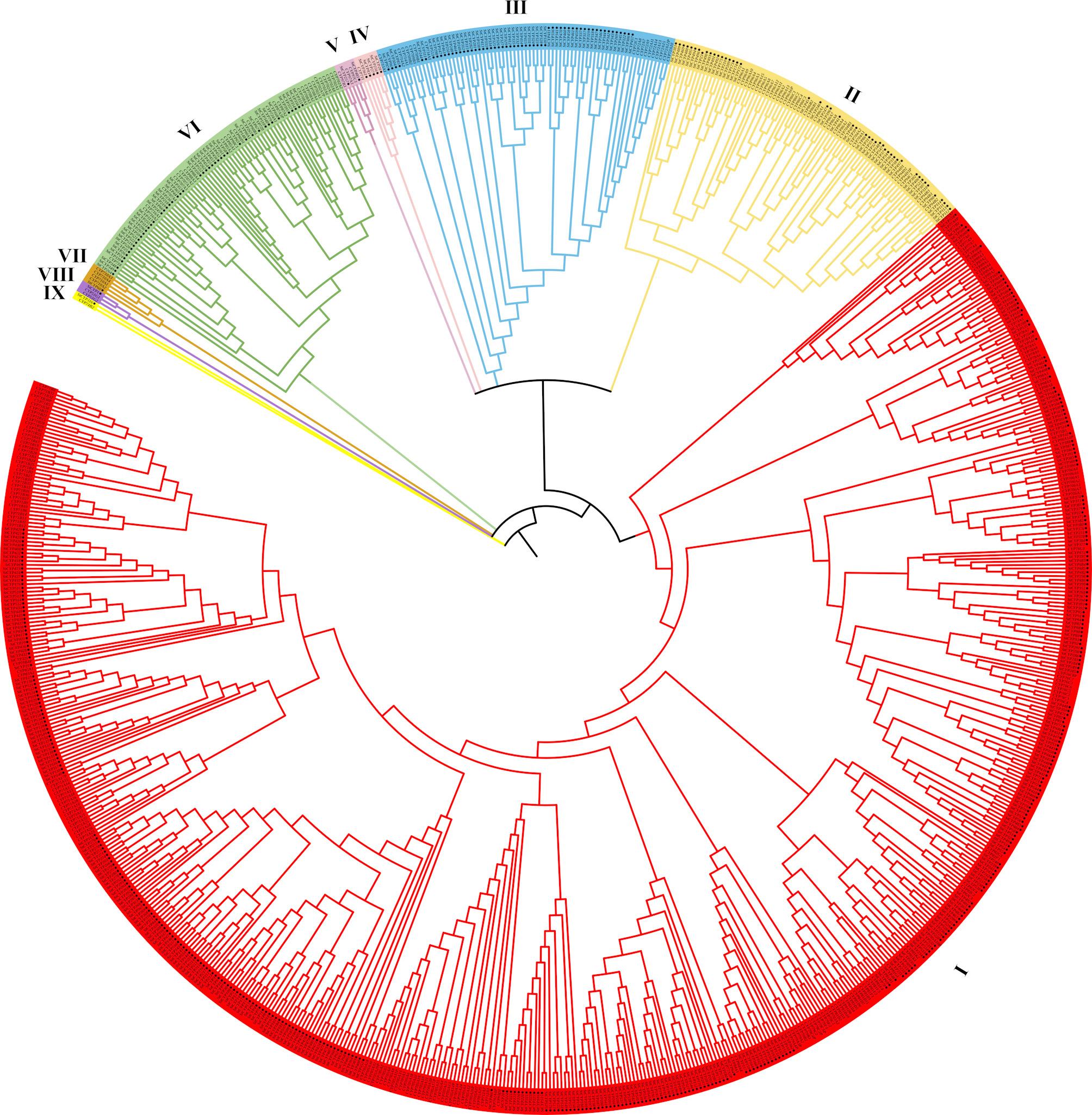
Phylogenetic tree analysis of CYP450 genes from potato and Arabidopsis. Ⅰ to Ⅸ represent different CYP clans: CYP71, CYP86, CYP72, CYP711, CYP97, CYP85, CYP710, CYP51, and CYP74 families, respectively. Sequences from potato are marked with a star (✹), while those from Arabidopsis are unmarked

To explore the evolutionary relationship of CYP450 genes, the protein sequences of 558 CYP450 genes from potatoes and 266 CYP450 genes from Arabidopsis were analyzed using MEGA5.05. The 558 StCYP450 proteins were classified into A-type and non-A-type. A-type (CYP71 clan) and non-A type (CYP72, CYP711, CYP97, CYP86, CYP710, CYP74, CYP85, and CYP51 clan) consisted of 395 and 163 StCYP proteins, respectively. The CYP71 clan was the most extensive, accounting for 70.8% (395/558) of the members. This distribution was similar to that observed in Arabidopsis (50% A type), tomato (58.80% A type), and sweet osmanthus (51.08% A type). The CYP71 clan could be further divided into 19 subfamilies (CYP71, CYP73, CYP75, CYP76, CYP77, CYP78, CYP79, CYP81, CYP82, CYP83, CYP84, CYP89, CYP93, CYP98, CYP701, CYP703, CYP705, CYP706, and CYP712). Furthermore, the CYP51, CYP74, and CYP710 clans each contained only one StCYP protein, while the CYP71 subfamily was the largest, with 120 members (Fig. 2). Briefly, different StCYP450 clans varied significantly in the number of members, and A-type CYP450 genes played a crucial role in the StCYP450 family.

### Cis-acting elements of the StCYP85 clan gene family in potato

**Fig. 3:**
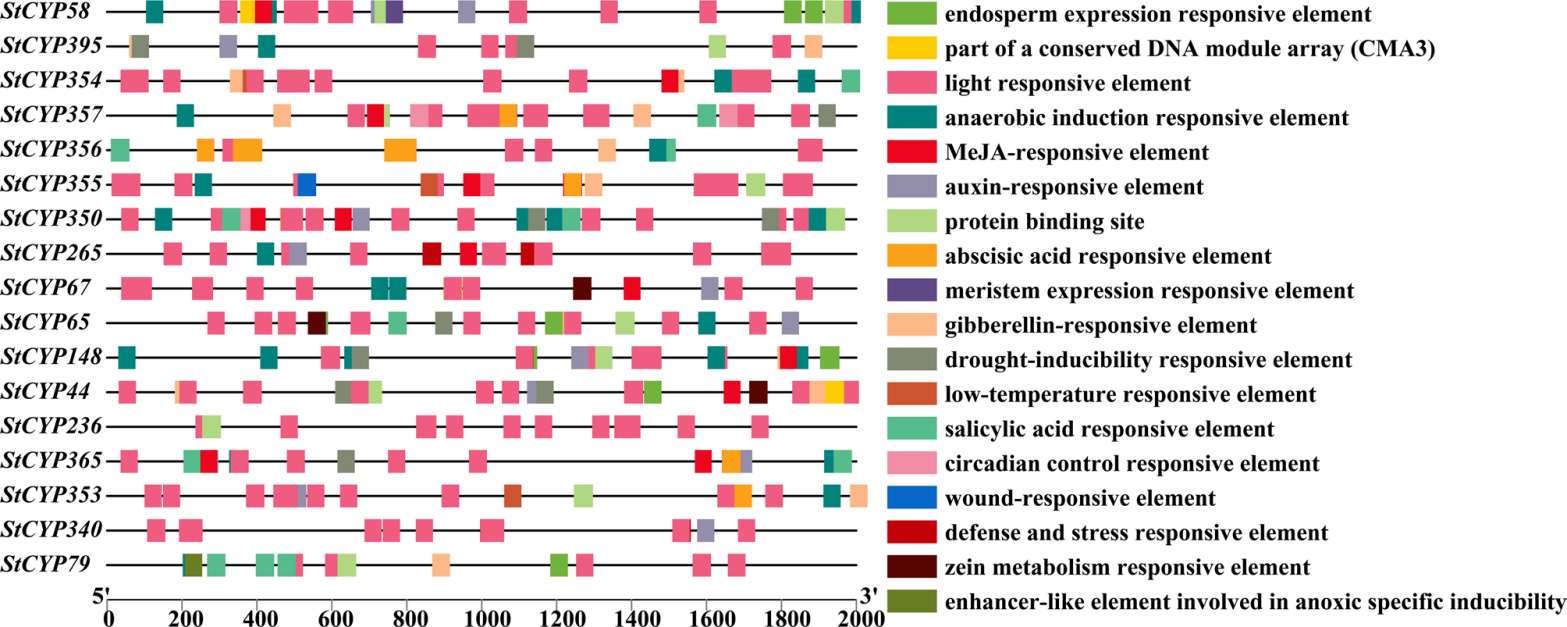
Analysis of *cis*-acting regulatory elements in promoters of the StCYP85 clan genes. The histogram shows the number of each type of *cis*-element identified in the promoters of 17 StCYP85 genes

*Cis*-acting elements play a key role in the transcription initiation factors and are critical for gene expression regulation [27]. To explore the roles of StCYP85 clan genes, 18 primary regulatory elements were scanned from 17 StCYP85 members. Six categories were identified, including light responsiveness elements (LRE, 1), phytohormone responsiveness elements (PRE, 5), stress responsiveness elements (SRE, 6), development-related (3), site-binding (2), and metabolism regulation (1) (Table 1; Fig. 3).

**Table 1:** Categorization and types of cis-regulatory elements (CREs) identified in the promoter regions of stcyps

SI. No	Element category	Number	cis-regulatory elements
1	Promoter related	9520	TGA-element, TGACG-motif, TGA-box, TCT-motif
			TC-rich repeats, TCCC-motif, TCA-element, TCA, TATC-box, TATA-box
2	Unknown	6138	-
3	Light-responsive	2714	MRE, MBSI, MBS, LTR, GATA-motif, GARE-motif, Gap-box
			GA-motif, F-box, motif I, ERE, E2Fb, E2F, DRE1
			DRE core, DRE, dOCT, CTAG-motif, W box
4	Hormone-responsive	704	WUN-motif, WRE3, W box, MYB, MSA-like, MRE
5	Abiotic and biotic stress	601	WUN-motif, WRE3, W box
6	Development-related	201	WUN-motif, TGA-element, MYB, MRE
7	Site-binding	193	WUN-motif

The StCYP450 promoter contained diverse *cis*-regulatory elements that were associated with light signal transduction, hormone response, and stress response. Many light response regulatory elements were present in StCYP450 genes, such as G-Box and TCT motif *cis*-elements. There were at least two box elements, and the *StCYP350* gene promoter contained nine elements. *StCYP236* contained the fewest stress-responsive elements, and no stress-responsive elements were detected in its promoter region. Among stress-related elements, many anaerobic induction elements (ARE), light responsive elements (LRE), drought induction (MBS), and abscisic acid (ABA) responsive elements (ABRE) existed in the *StCYP85* gene promoter regions. The *StCYP148* and *StCYP350* gene promoter regions contained the highest number of ARE elements, with five elements each. Additionally, 22 LRE and 13 ABRE elements were found in *StCYP356*. Furthermore, eight genes contained 1–2 MBS elements. These findings implied that StCYP450 genes may be crucial in the light, hormone, and stress responses of potato plants through various *cis*-acting elements.

### Protein interaction network and functional annotation of StCYP450 related to plant hormones in potato

**Fig. 4:**
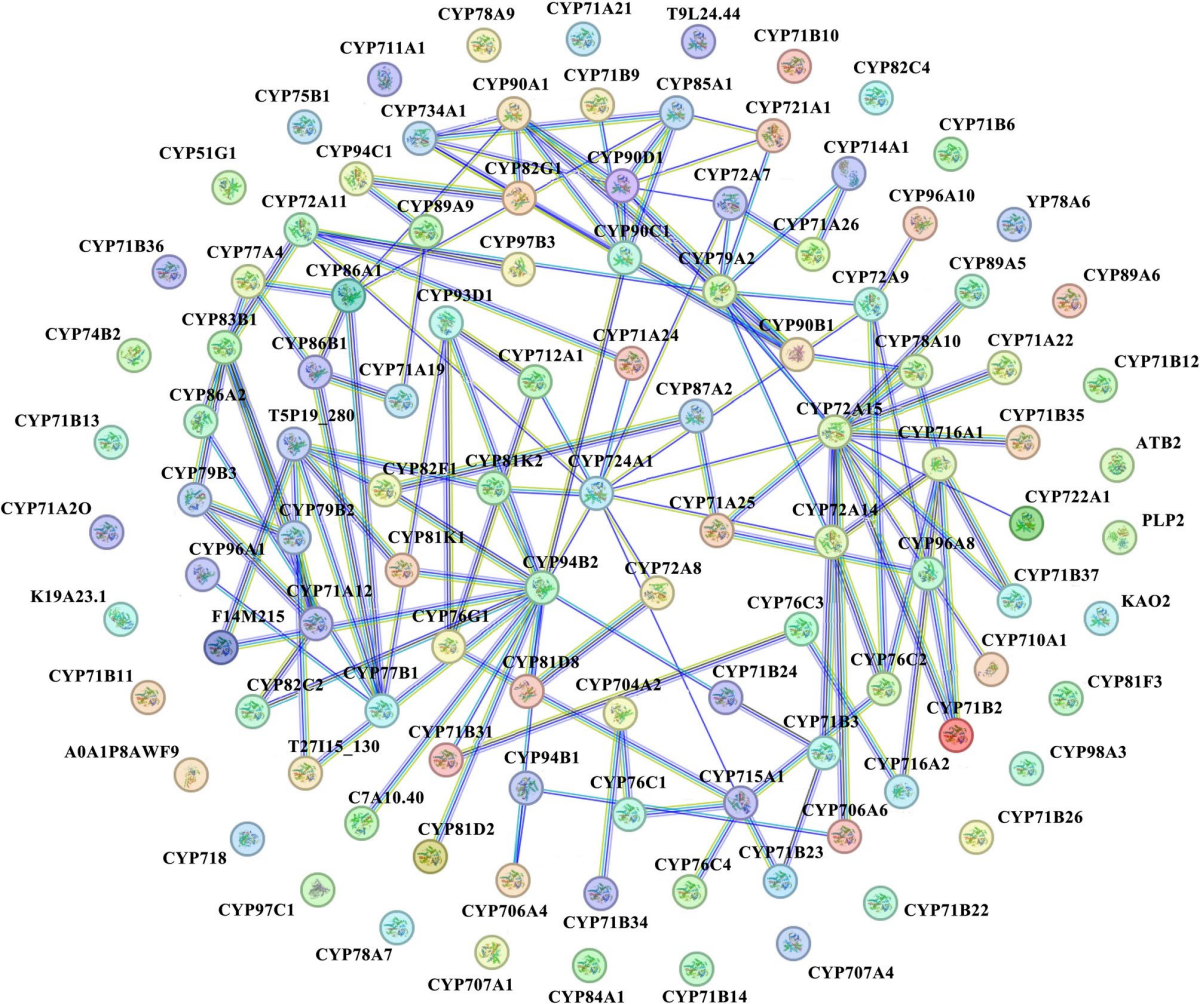
Protein-protein interaction (PPI) network of StCYP450 proteins. Nodes represent proteins, and edges indicate predicted functional associations. Colored clusters represent groups of proteins with stronger interactions, suggesting potential involvement in common biological pathways, including brassinosteroid biosynthesis and abiotic stress response

Previous studies have indicated that many CYPs participated in BRs synthesis and played an important part in responding to biotic and abiotic stress [10, 28–30]. To further understand the role of StCYP450s, we explored the interaction between CYP450 proteins that might participate in BRs biosynthesis and abiotic stress response. Using the model plant Arabidopsis as a background, 110 potential interacting proteins directly related to StCYP450 proteins were identified (Fig. 4 and Table S2). Among these, nine StCYP450s were involved in BRs biosynthesis and homeostasis (mainly including *StCYP58*, *StCYP65*, *StCYP67*, *StCYP147*, *StCYP148*, *StCYP248*, *StCYP250*, *StCYP298* and *StCYP553*). The *StCYP58*, *StCYP65*, *StCYP67* and *StCYP148* were involved in BRs biosynthesis, and *StCYP65* was also essential for the regulation of leaf cell polar elongation. Other StCYPs were involved in BRs inactivation and regulation of homeostasis. Four StCYP450s (*StCYP44*, *StCYP236*, *StCYP353* and *StCYP365*) participated in the oxidative degradation of ABA and were important for regulating ABA levels (Tables S3).

### Functional annotation of StCYP85 clan genes in potato

**Fig. 5:**
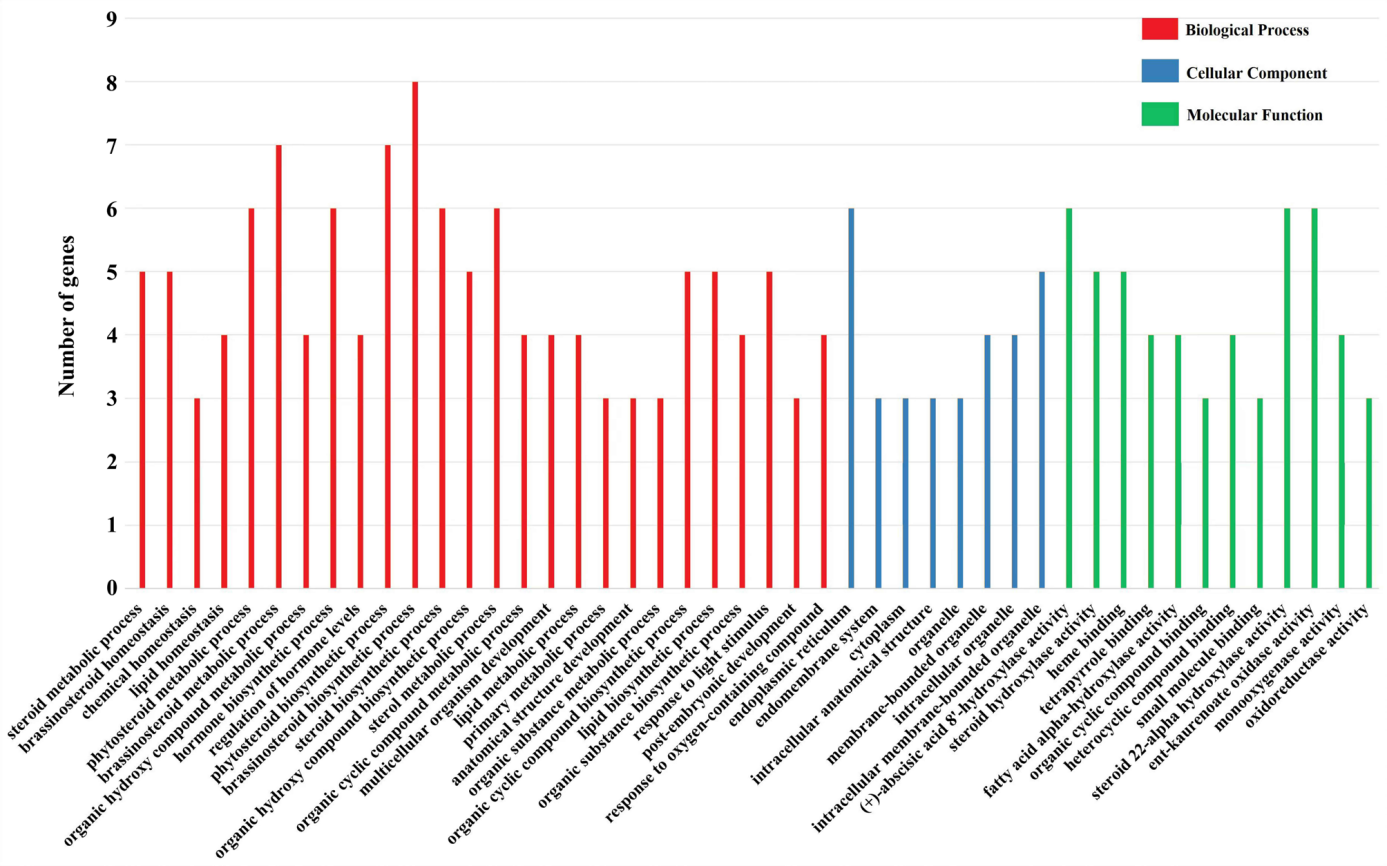
GO annotation of StCYP85 clan genes in potato. The y-axis indicates the number of genes annotated to each specific GO term. Key enriched terms include brassinosteroid metabolic process, intracellular membrane-bounded organelles, and steroid hydroxylase activity

The function of StCYP85 clan genes was annotated against the Gene Ontology (GO) databases. As shown in Fig. 5 and Table S4, StCYP85 clan genes were classified into 46 functional groups, belonging to ‘Biological Process, BP’ (26), ‘Cell Component, CC’ (8) and ‘Molecular Function, MF’ (12). In BP, 19, 19, and 17 StCYPs were identified to be involved in BRs homeostasis (GO: 0010268), BRs metabolic process (GO: 0016131) and BRs biosynthetic process (GO: 0016132), respectively. In addition, *StCYPs* participated in the response to biological stimulus (GO: 0009416). In CC, *StCYP67* and *StCYP350* were localized to the cytoplasm (GO: 0005737), intracellular organelle (GO: 0043229) and intracellular membrane-bounded organelle (GO: 0043227 and GO: 0043231). These results showed high consistency with subcellular localization prediction. In MF, *StCYPs* were primarily associated with steroid hydroxylase (GO: 0008395 and GO: 0010012) and compound binding (GO: 0020037, GO: 0046906, GO: 0097159, GO: 1901363).

### Subcellular localization assay of StCYP67

**Fig. 6:**
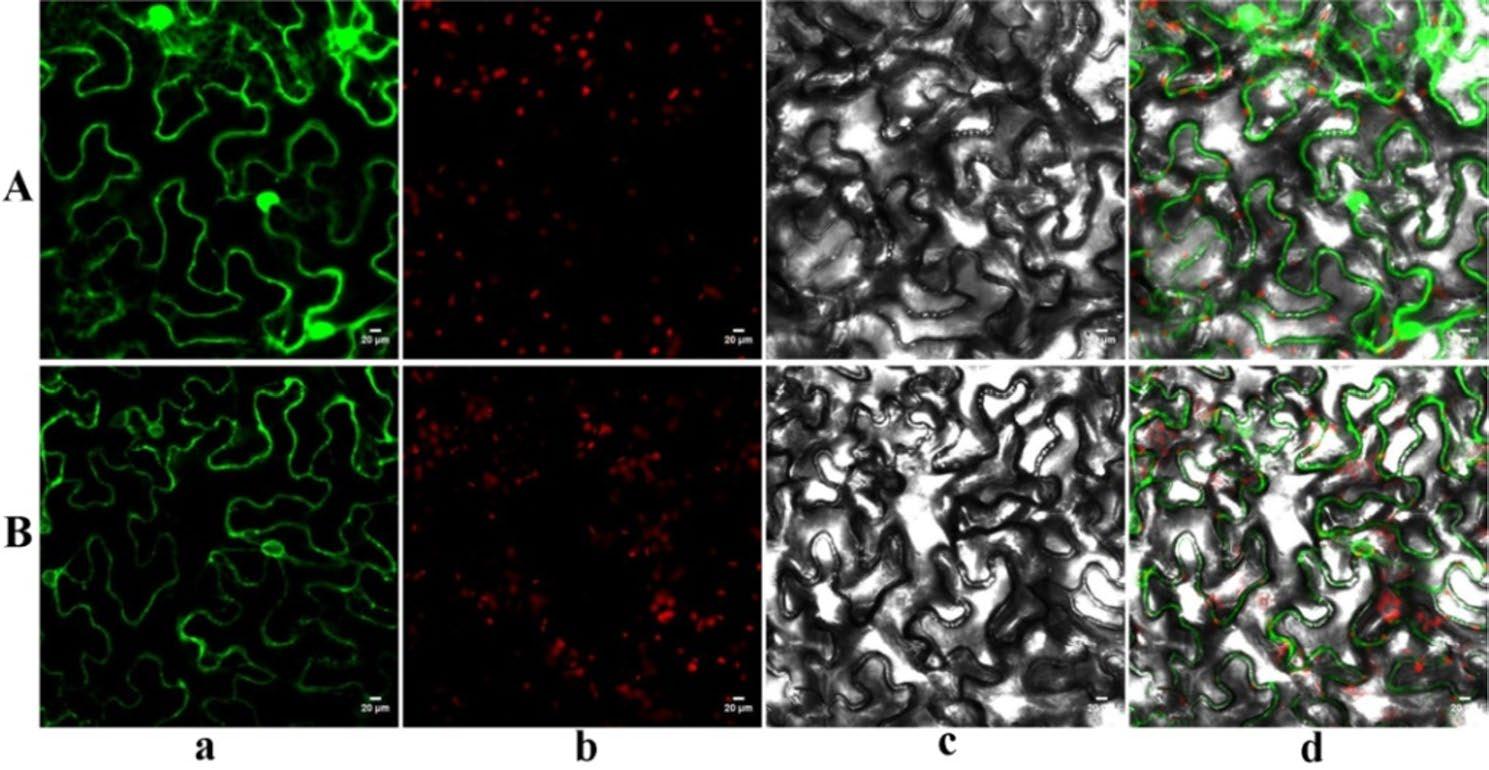
Subcellular localization of StCYP67 in tobacco epidermal leaf cells. Control expression of empty pBWA(V)HS-GFP vector (**A**). Localization of the StCYP67-GFP fusion protein (**B**). **a** GFP fluorescence channel. **b** Chloroplast fluorescence channel. **c** Bright field, and **d** Merged field. The fusion protein StCYP67-GFP and the control vector were transiently expressed in tobacco leaves and then observed by fluorescence microscopy. Scale bar, 20 μm

The green fluorescent protein (GFP) vectors were introduced into tobacco leaves, and the fluorescence was visualized using laser scanning confocal microscopy. The StCYP67-GFP fusion proteins were detected in the endoplasmic reticulum, aligning with the predicted results (Fig. 6). This indicated that StCYP67 is predominantly located in the endoplasmic reticulum.

### Effects of Cd^2+^ stresses on phenotypes of potatoes

**Fig. 7:**
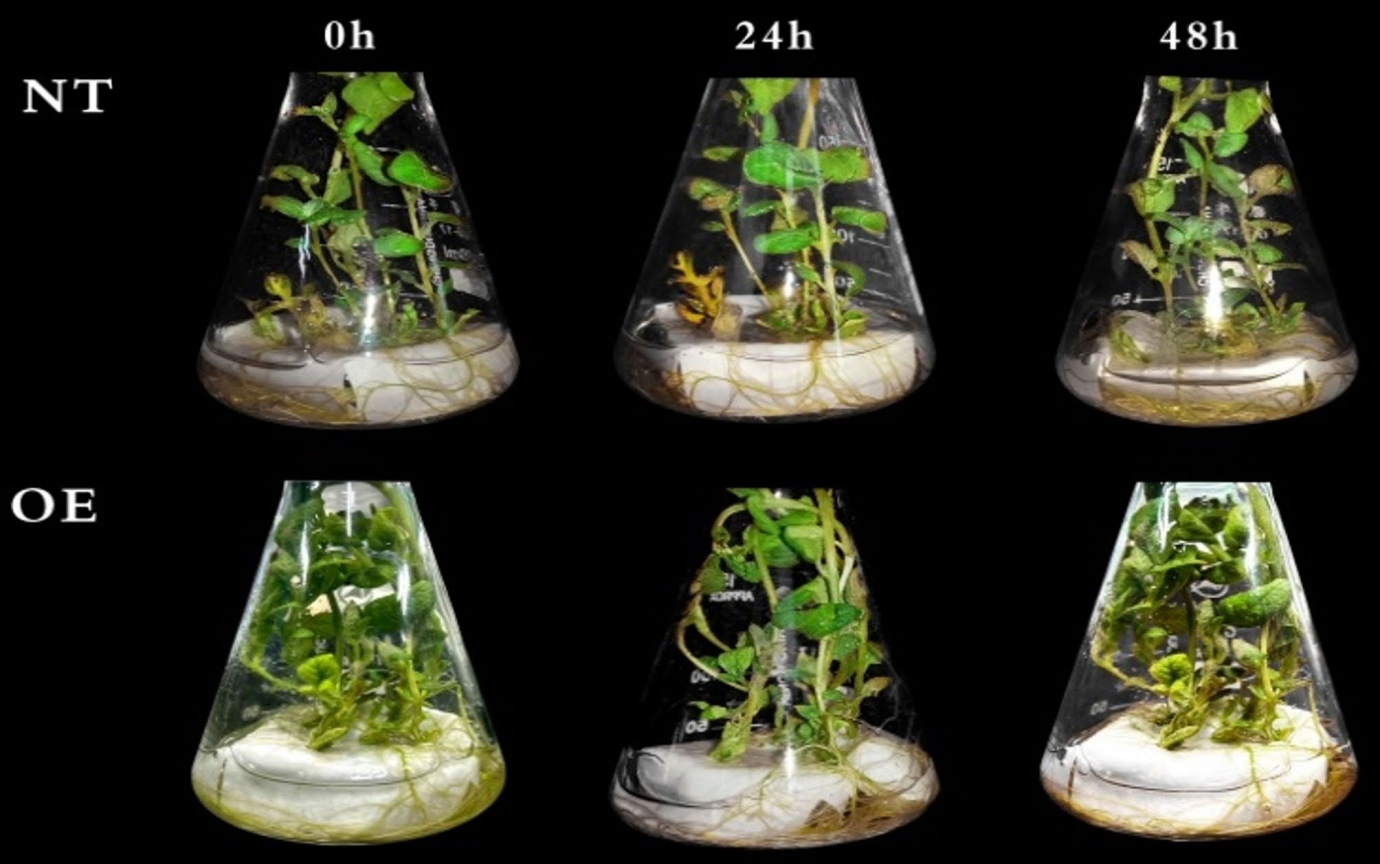
Phenotypic response of OE and NT potatoes cultured for 0 h, 24 h and 48 h under Cd^2+^ stresses

As shown in Fig. 7, the phenotypic variations in NT and OE potato seedlings were monitored after treatment with 100 µM CdCl_2_·2.5 H_2_O for 0 h, 24 h and 48 h. When uniformly grown seedlings were cultivated for 30 d, transgenic plants appeared thicker and stronger than NT. This indicated that *StCYP67* promoted the growth of potatoes. Under heavy metal stress, transgenic lines exhibited greater tolerance compared to NT plants. In contrast to the OE plants, Cd^2+^ caused the NT leaves to slightly curl at 24 h, and the NT leaves became wilted and shrunken under 48 h heavy metal stresses [31].

### Analysis of StCYP85 and StCYP72 gene expression in different tissues and treatments in DM potato

**Fig. 8:**
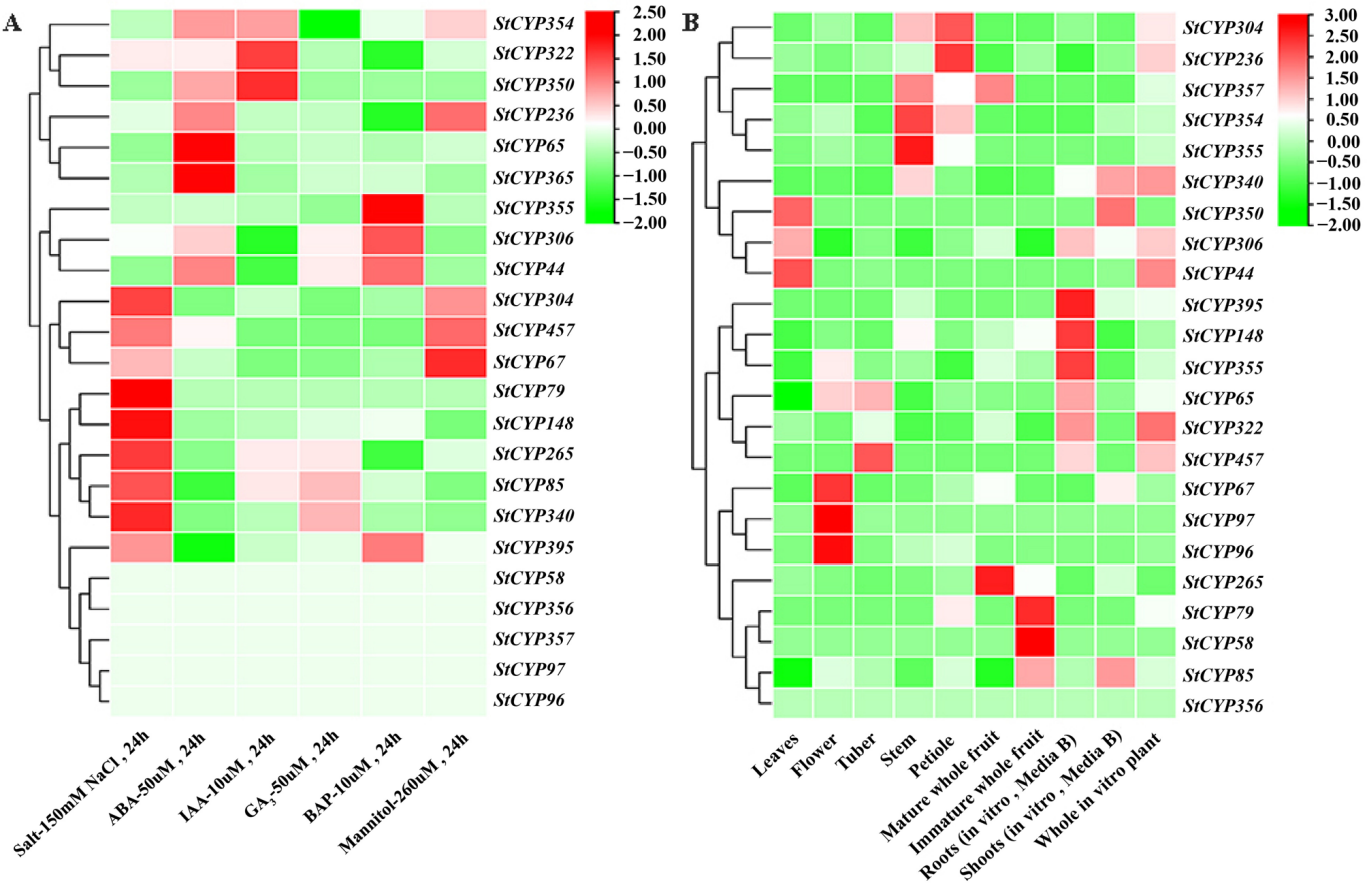
Expression profiles of StCYP72 and StCYP85 genes. (**A**) Heatmap of gene expression under various abiotic stress and hormone treatments at 24 h. (**B**) Tissue-specific expression patterns across ten different potato organs. Expression values are FPKM values from RNA-seq data The color scale represents expression levels from low (green) to high (red). Data represent the mean of three biological replicates

To further understand the function of StCYP450 genes, the expression levels of 16 StCYP85 and seven StCYP72 genes in ten different tissues were quantified. These tissues included tubers, leaves, flowers, stems, shoots, roots, petioles, mature whole fruit, immature whole fruit, and whole in vitro plants. The analysis was based on the FPKM values (Table S5) from the RNA-seq data of the DM potato database. The results indicated that all of StCYP85 and StCYP72 genes were expressed in these ten tissues. As shown in Fig. 8B, the expression levels of StCYP85 and StCYP72 genes varied significantly across different tissues, suggesting functional diversification among StCYP genes. For instance, *StCYP65* and *StCYP457* were expressed exclusively in tubers, while *StCYP265* and *StCYP 357* were expressed exclusively in roots. *StCYP67* was upregulated in most tissues, especially in flowers and shoots.

Additionally, we analyzed the expression of StCYP genes under various abiotic treatments at 24 h (Table S6, Fig. 8A). The StCYP genes exhibited diverse expression patterns. We found that three StCYP genes (*StCYP67*, *StCYP304* and *StCYP457*) were consistently highly expressed under NaCl or mannitol treatment. The expression of *StCYP65*, *StCYP67*, *StCYP85*, and *StCYP306* was upregulated following bone alkaline phosphatase (BAP) treatment. Under ABA treatment, nine members showed upregulation, whereas three members were downregulated in response to gibberellic acid (GA_3_) treatment. Notably, five StCYPs (*StCYP65*, *StCYP67*, *StCYP85*, *StCYP236* and *StCYP306*) were highly expressed, whereas six StCYPs (*StCYP58*, *StCYP79*, *StCYP96*, *StCYP97*,* StCYP356* and *StCYP357*) showed no expression under all of the above abiotic treatments.

### Expression of stcyp genes under Cd^2+^, ABA and EBL treatments

**Fig. 9:**
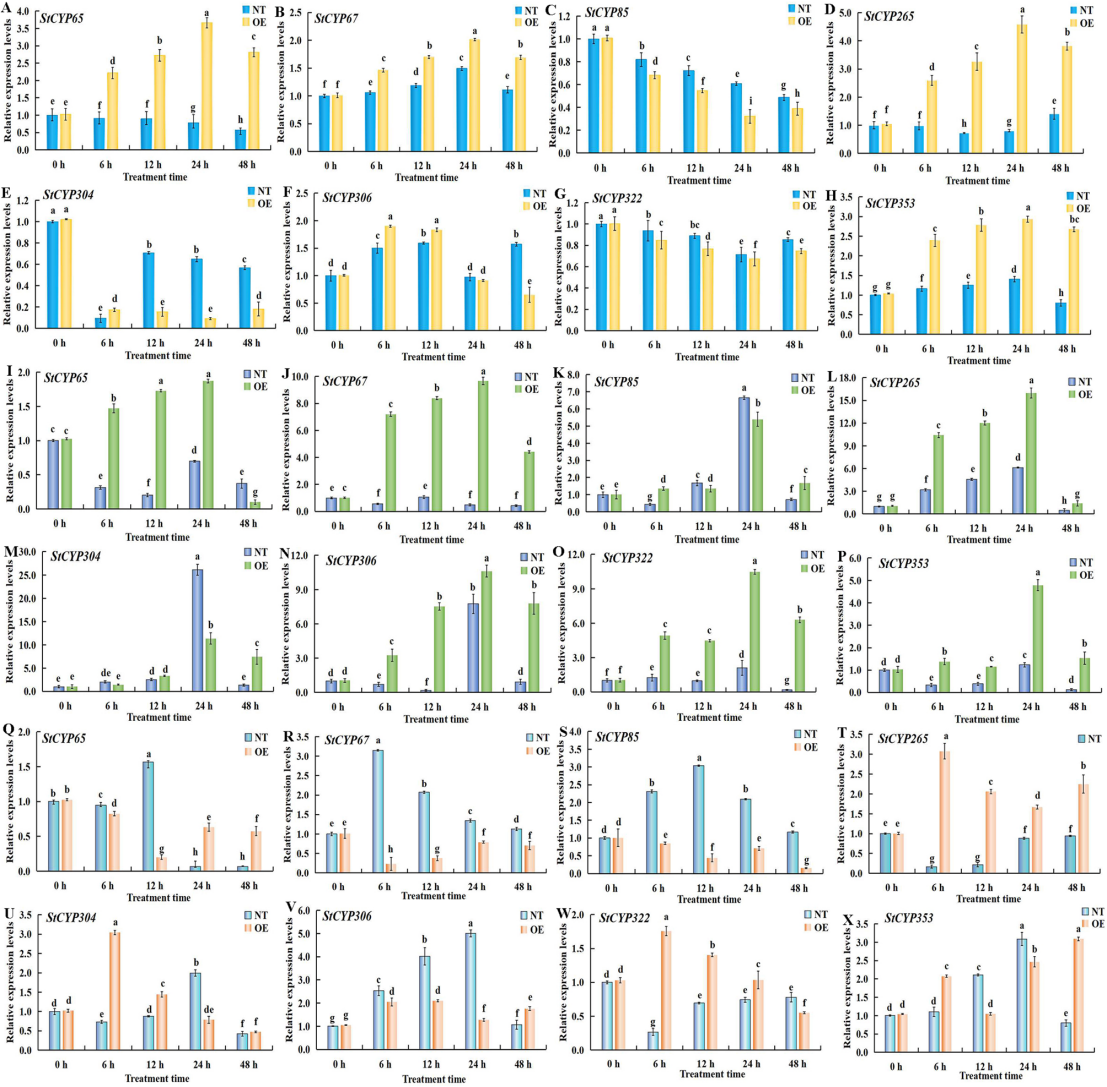
Quantitative RT-PCR analysis of eight StCYP genes under Cd^2+^, ABA, and EBL treatments. (**A**-**H**) Expression changes under Cd^2+^ stress. (**I**-**P**) Expression under ABA treatment. (**Q**-**X**) Expression under EBL treatment. The relative expression was normalized to *Stef1α* and calibrated to the 0 h control. Data are presented as mean ± SD (*n* = 3). Different letters indicate statistically significant differences between means (*P* < 0.05, one-way ANOVA)

To further confirm the expression levels of eight candidate StCYP genes under Cd^2+^ stress, ABA and exogenous EBL treatment in potatoes, qRT-PCR was performed (Fig. 9).

After treatment with 100 µM CdCl_2_·2.5H_2_O, *StCYP65*, *StCYP67*, *StCYP265* and *StCYP353* exhibited similar expression patterns in OE plants. The highest expression levels were observed at 24 h post-treatment. However, in NT plants, only *StCYP353* showed a similar trend, while *StCYP265* displayed an opposite trend. *StCYP85*, *StCYP322*, and *StCYP304* showed similar expression patterns in both OE and NT plants. Notably, the highest expression level of *StCYP306* occurred at 6 h in OE plants and at 12 h in NT in response to Cd²⁺ stress.

We noted that most StCYP genes were induced and varied at different treatment times. Compared to the controls, all eight StCYP genes were significantly upregulated in OE potatoes at 24 h under medium ABA treatment. In particular, *StCYP265* and *StCYP304* showed significantly higher expression levels under ABA treatment. However, *StCYP65* and *StCYP67* showed downregulation in NT potatoes under ABA treatments.

Following EBL treatment, the expression levels of some StCYP genes were enhanced. They peaked at 6 h (*StCYP67* in NT, *StCYP265*, *StCYP304* and *StCYP322* in OE), 12 h (*StCYP65* and *StCYP85* in NT), or 24 h (*StCYP306* and *StCYP353* in NT). Interestingly, the expression levels of *StCYP265* and *StCYP304* in OE plants increased initially and then decreased, peaking at 6 h. In contrast, *StCYP304* showed an opposite trend in NT plants. Meanwhile, the expression levels of *StCYP265* in NT plants and *StCYP65*, *StCYP67*, and *StCYP85* in OE plants were downregulated at all EBL treatment times.

These results indicated that numerous StCYP genes likely served critical functions in potato responses to abiotic stress responses and in mediating ABA, BRs signaling pathways.

### Expression of StCYP85 genes in StCYP67-overexpressed potato under heavy metal stress based on transcriptomics

**Fig. 10:**
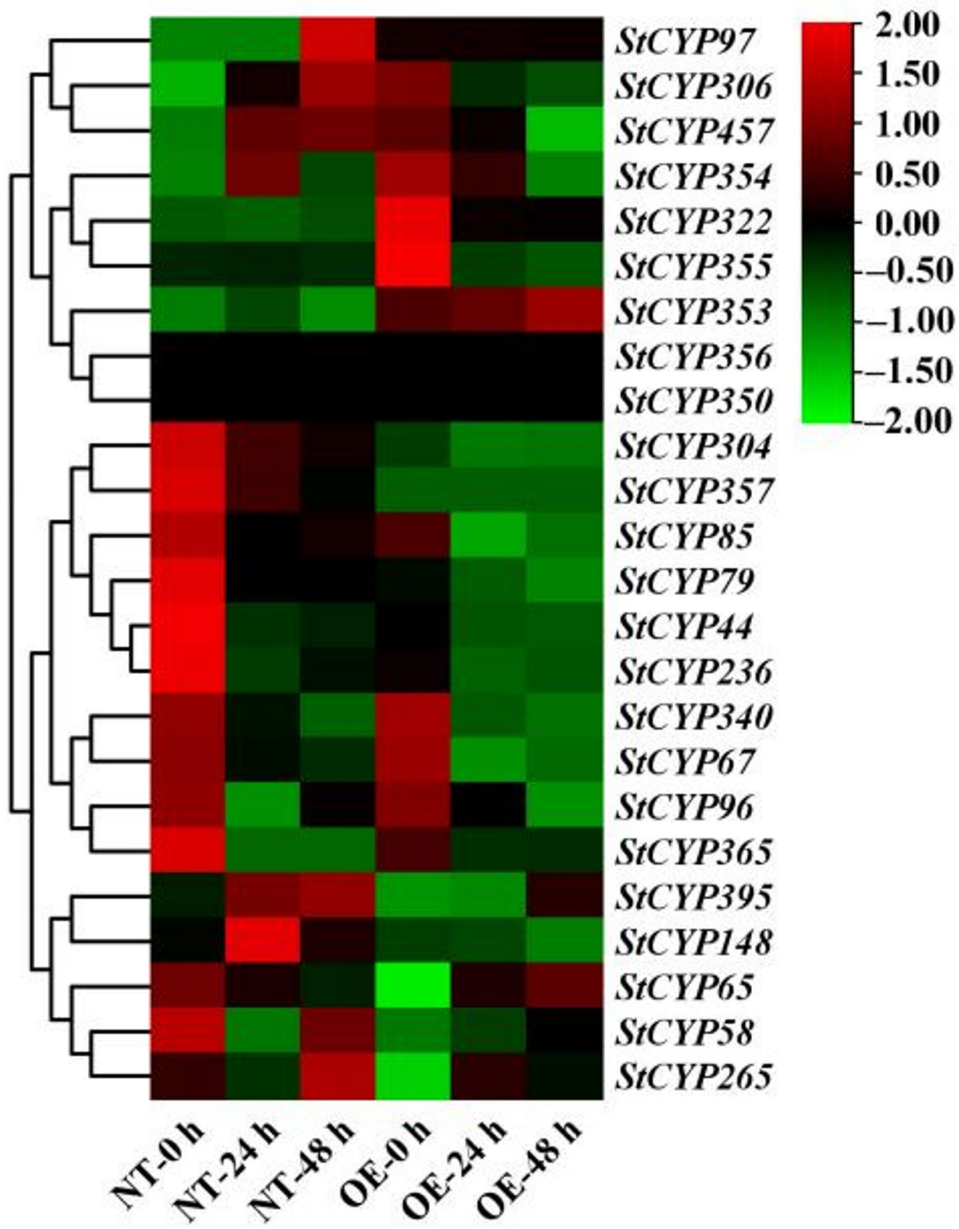
StCYP85 genes expression pattern in OE and NT potatoes under Cd^2+^ stress based on transcriptome analysis

To investigate the potential functions of the candidate StCYPs in resisting heavy metal stress, transcriptome analysis of OE and NT potatoes was conducted under 100 µM CdCl_2_ · 2.5 H_2_O (Fig. 10). The transcriptome data have been published and were validated for accuracy.

The expression levels of 24 *StCYP85* genes were analyzed. Most of these genes responded to heavy metal stress at different treatment times. Nine StCYP genes (*StCYP85*, *StCYP96*, *StCYP306*, *StCYP322*, *StCYP340*, *StCYP354*, *StCYP355*, *StCYP365* and *StCYP457*) in OE were significantly down regulated under moderate heavy metal stress (24 h). In contrast, three StCYP genes (*StCYP65*, *StCYP353* and *StCYP395*) were upregulated under severe heavy metal stress (48 h) compared to NT plants. Notably, *StCYP322 and StCYP355* were upregulated in OE plants. *StCYP322* showed the highest expression level in OE plants (3.5-fold increase over controls), whereas *StCYP355* exhibited the lowest expression (1.9-fold increase).

*StCYP395* and *StCYP265* were significantly upregulated under different levels of treatment in both OE and NT plants. These observations demonstrated that StCYP genes may be involved not only in responses to salt stress and moderate heavy metal stress but also play a role in severe heavy metal stress (Figs. 8A and 10). Furthermore, the qRT-PCR results were consistent with the RNA-seq data, confirming the reliability of the RNA-seq online data.

## Discussions

### Evolutionary analysis and classification of StCYP450 genes

As one of the largest gene families, the StCYP450 gene family has been identified to be vital in various plant metabolic processes, development and stress responses [32–34]. In this study, we identified 558 CYP450 members in potato, a number substantially higher than those reported in Arabidopsis (245) [3] and tomato (233) [35]. Evolutionary relationships and functional predictions indicated a closer genetic affinity between potato and tomato compared to Arabidopsis. Further analyses of chromosomal distributions, gene architectures, conserved protein motifs, and gene duplication events indicated that StCYP450 genes within the same phylogenetic clade shared conserved features.

Exon-intron structures play a crucial role in gene evolution and phylogenetic analysis [36, 37]. Our structural analysis of StCYP450s supports this notion. Notably, CYP51, CYP710, and CYP85 exhibit strong evolutionary connections, suggesting a common ancestral origin related to sterol metabolism. Additionally, CYP85, CYP724B, and CYP90 have a crucial function in the biosynthesis of BRs and are closely related. Specifically, CYP724B and CYP90B are phylogenetically adjacent and both are implicated in regulating C-22 hydroxylation during BRs synthesis [38].

Promoter activity is essential for regulating gene function [39]. *Cis*-regulatory elements analysis revealed that *StCYP450* promoters are enriched in elements related to phytohormone modulation, stress response, and light reaction. These findings support the broad functional importance of StCYP450 genes in regulating potato growth regulation and developmental processes.

### Functions analysis of StCYP450 genes in BRs synthesis

GO analysis revealed significant functional enrichment of StCYP450 genes across BP, CC and MF, indicating their diverse roles. Similar results have been reported for the CYP450 gene families in Arabidopsis and *Sorghum bicolor* L [35, 40]., underscoring the conserved regulatory roles of CYP450 genes across plant species.

StCYP450s were found to be predominantly expressed in stems and roots, suggesting a specialized function of *StCYP67* in transgenic potatoes. The tissue specificity of StCYP450 genes implies functional diversification among StCYP450 members in growth and development regulation. For instance, tissue-specific expression has also been observed for CYP450 genes in maize (*Zea mays* L.) and Chinese cabbage (*Brassica rapa* L. ssp. *pekinensis*) [41, 42], supporting the notion that CYP450s contribute to tissue-specific physiological processes. This aligns with previous studies demonstrating that CYP450 members are widely expressed and participate in secondary metabolite synthesis [43]. For example, *StCYP90A* played an essential role in BRs synthesis and was found to be more highly expressed in leaves than in other tissues [44]. Similarly, *StCYP67* was highly expressed in roots, supporting the hypothesis that certain StCYP450 genes displayed tissue-preferential expression profiles and may play a significant role in BRs synthesis.

Notably, *StCYP265* was highly expressed only in control conditions but not in EBL-treated potatoes. This indicated its potential role in the synthesis and signal transduction of BRs. Similarly, *StCYP65*, which was downregulated in ABA-treated potatoes, may also contribute to BRs biosynthesis. Both BRs and ABA are important in resisting abiotic stresses. Previous studies have shown that ABA-upregulated genes can be suppressed by BRs under salt stress [45, 46]. In tomato, CYP90B3 and CYP724B2 families are involved in BRs biosynthesis [10]. Analogously, *StCYP265*, *StCYP304* and *StCYP306* may contribute to BRs synthesis, signal transduction, and protection against various abiotic stresses, as indicated by their elevated expression under ABA treatment.

### Expression of StCYP450 genes under abiotic stresses and hormone treatments

CYPs are known to protect plants against environmental stresses [47–49]. The expression of StCYP450 genes was investigated under Cd^2+^, ABA and EBL treatments. Stress-responsive gene expression exhibited stimulus-dependent variation, consistent with previously reported context-specific transcriptional regulation [50].

Under Cd^2+^ stress, differential expression of StCYP genes in OE versus NT plants suggested their involvement in a coordinated detoxification or stress adaptation mechanism. They may be involved in detoxification or metabolic pathways activated under such conditions. In contrast, *StCYP85* displayed a consistent expression pattern in both OE and NT plants, indicating a generalized response to cadmium. A particularly notable observation was the distinct temporal expression peak of *StCYP306* in OE plants, suggesting genotype-specific regulation that may enable earlier or more efficient detoxification. This could reflect differences in heavy metal perception or signaling between OE and NT plants. In conclusion, these findings reveal complex and diverse expression patterns of CYP genes during Cd^2+^ exposure, with both conserved and genotype-specific responses. These results align with Mehrian et al., who documented marked induction of *CYP71C1*, *CYP71C3v2*, and *CYP81A9* and suppression of *CYP72A5* in maize under Cd^2+^ stress [51].

CYP450 genes are also critically important in responding to ABA treatment. Studies in pear have demonstrated marked ABA-mediated upregulation of *CYP450*s [52], and tomato CYP450 genes are upregulated under ABA, cold, drought, NaCl, and heat treatments [53]. Most StCYP genes were induced by ABA in a time-dependent manner, indicating a dynamic regulatory role. Integrating these results. we propose that these genes regulate ABA levels and enhance abiotic stress tolerance in potato plants. It has been reported that ABA regulates numerous BR-responsive genes, and BRs can also induce some key ABA- and stress-responsive genes [45, 54].

Interestingly, several StCYP genes displayed consistent downregulation across all EBL treatment durations. This repression may reflect direct inhibition by EBL or dominant secondary effects overriding inductive signals. Alternatively, this may represent a compensatory mechanism to maintain cellular P450 homeostasis, particularly if these genes function in pathways not directly promoted by EBL. These observations underscore the complexity of EBL-mediated regulation, involving both temporal and response specificity. Previous studies have shown that EBL modulates CYP expression in maize, activating enzymatic and non-enzym antioxidant systems to scavenge ROS and reduce oxidative damage [51].

Together, these results enhance our understanding of the regulatory mechanisms underlying plant stress adaptation and detoxification. They also identify promising targets for genetic engineering aimed at enhancing stress resistance in crops. Further studies should prioritize functional characterization of StCYP450 genes to elucidate their roles in cadmium detoxification, EBL and ABA signaling, and metabolic pathways, as well as the mechanisms driving their expression patterns.

## Conclusion

In this study, 558 *StCYP450* genes were evaluated, and their structures and functions were characterized. Functional annotation through GO enrichment highlighted the pivotal roles of StCYP450 family members in transcriptional regulation and responses to abiotic stresses. Spatial expression profiling demonstrated ubiquitous transcriptional activity across multiple tissue types, except in shoots. Ultimately, our functional characterization of *StCYP67* demonstrated its potential as a key regulator in balancing Cd^2+^ tolerance and phytohormone signaling, highlighting its value as a candidate gene for breeding stress-resistant potatoes. This study provides a foundation for investigating the functional genomics of potato CYP450s. The results further provide critical molecular resources for developing stress-resilient potato cultivars through targeted breeding strategies.

## Materials and methods

### Identification of StCYP450 gene family in potato

The potato whole genome data, CDS sequences, protein sequences and annotation files were obtained from the Spud DB Potato Genomics Resource (http://solanaceae.plantbiology.msu.edu/) [55]. We identified CYP450 sequences using a Hidden Markov Model (HMM) [56] and the Basic Local Alignment Search Tool (BLAST) [57]. The CYP450 conserved structure domain HMMER file (PF00067) as the query sequence was downloaded from Pfam (http://pfam.xfam.org/) [58]. Initial identification of *StCYP450* candidates was performed using HMMER 3.0 with an expected value (E-value) of 1e-5 followed by domain validation through sequential analysis with pfamscan (https://www.ebi.ac.uk/Tools/pfa/pfamscan/) [59], NCBI-CDD (https://www.ncbi.nlm.nih.gov/Structure/bwrpsb/bwrpsb.cgi), Inter Pro (http://www.ebi.ac.uk/interpro/scan.html) and SMART (http://smart.embl-heidelberg.de/) [60–63]. Final gene family members were systematically annotated through consultation with the Cytochrome P450 Nomenclature Committee (David Nelson: dnelson@uthsc.edu) [1]. Arabidopsis CYP450 ortholog sequences were obtained from TAIR (https://www.arabidopsis.org/). Subcellular localizations of StCYP450 proteins were predicted via WoLF PSORT (https://wolfpsort.hgc.jp/) [64], while biophysical parameters including molecular weight and isoelectric points were calculated using ExPASY (https://web.expasy.org/compute_pi/) [65].

### Gene structures and conserved motifs of StCYP450 proteins

Conserved motifs of StCYP450 proteins were predicted using the MEME Suite web server (https://meme-suite.org/meme/) [66], in which the Maximum number of motifs was 10 and all other parameters were defaulted, and the mast XML file was downloaded. Based on the annotated information of *GFF3* gene structure, the conserved motifs and gene structures were visualized and analyzed by TBtools software (v2.007, https://tbtools.cowtransfer.com/s/0a9cbf41b47b4a) [67].

### Chromosomal mapping analysis of StCYP450 genes

Physical chromosomal coordinates for StCYP450 loci were retrieved from the Spud DB Potato Genomics Resource database [68], enabling systematic mapping of gene positions across the potato genome.

### Evolutionary relationships in CYP450 gene clusters

Comparative genomics analyses were conducted using MCScanX to delineate both segmental duplication events and conserved collinear relationships between potatoes, Arabidopsis, and tomatoes [69], and the covariance results were visualized using TBtools.

### Phylogenetic linkages in CYP450 gene families

To resolve evolutionary linkages between potato and Arabidopsis CYP450 homologs, protein sequences were retrieved from the Ensembl plants database (https://plants.ensembl.org/index.html) and aligned using MEGA-X with ClustalW. Phylogenetic reconstruction was performed using neighbor-joining methodology with 1000 bootstrap iterations to assess nodal robustness [70]. The evolutionary tree topology of StCYP450 with other plant CYP450 was visualized and annotated on the iTOL (https://itol.embl.de/) online website.

### Cis-acting elements analysis of StCYP85 genes

Upstream regulatory regions (2 kb proximal to translation initiation sites) of StCYP85 clade members were interrogated using PlantCARE (http://bioinformatics.psb.ugent.be/webtools/PlantCA-RE/html/). The types, numbers and functions of *cis*-acting elements of *StCYP450* gene promoters were predicted and visualized by TBtools, enabling systematic cataloging of *cis*-regulatory motifs implicated in transcriptional control mechanisms [71].

### Protein interaction of CYP450 in potato

Using the model plant Arabidopsis as a reference, protein-protein interaction networks for StCYP450s were computationally predicted using the STRING (https://cn.string-db.org/) [72].

### Functional annotation of StCYP450 genes

The functional classification and annotation of StCYP450 genes were performed using the eggNOG-mapper website (http://eggnog-mapper.embl.de/) [73].

### Subcellular localization of StCYP67 protein

The subcellular localization of StCYP67 was predicted to be at the endoplasmic reticulum. To validate this localization, the entire coding sequence of the *StCYP67* gene was amplified from potato using gene-specific primers (Table S7). The default cycling conditions (one cycle of 5 min at 94 °C; 30 cycles of 30 s at 94 °C, 45 s at 50 °C, 88 s at 72 °C; and 10 min at 72 °C) were carried out in a thermal cycler (BIO-RAD PCR System, T100TM). The cloned *StCYP67* gene was subsequently fused with a green fluorescent protein (GFP) expression vector pBWA(V)HS-ccdb-GLosgfp, using BioRun Seamless Cloning Kit (#RDA01) (Biorun, Wuhan, China). The constructed plasmids were then electrotransformed into *Agrobacterium tumefaciens* carrying the pBWA (V) HS-ccdb-GFP expression vectors. Tobacco seedlings were cultured for 30 d in a greenhouse before being subjected to low Light conditions for an additional 2 d to reduce chlorophyll interference. Subcellular distribution patterns of StCYP67 were visualized using laser confocal microscopy (Nikon C2-ER, Tokyo, Japan) [74]. The experiment included three biological replicates. Images from a representative experiment are shown.

### Expression of StCYP450 gene in different tissues and different treatments in DM potato

RNA sequencing data of DM potatoes under different tissues and treatments were publicly available and downloaded.

### Materials and treatments

The *StDWF4* gene over-expression in the potato cultivar ‘Zihuabai’ (OE) and nontransgenic potato plants (NT) were used as experimental materials [13]. Seedlings were planted in liquid Murashige-Skoog (MS) medium, 16 h light/8 h dark, at 25 °C and 80% relative humidity (RH). After 30 d of growth, evenly growing plants were selected for abiotic treatments [75]. For heavy metal stress, the Liquid medium was replaced with a Liquid MS medium containing 100 µM CdCl_2_·2.5H_2_O, with plain Liquid MS medium serving as the control. For ABA or BRs treatment, plant leaves were evenly sprayed with 100 µM ABA or 10 µM EBL solutions (freshly prepared) [76]. Leaf tissues were harvested at 0 h, 2 h, 6 h, 12 h, 24 h and 48 h post-treatment, flash-frozen in liquid nitrogen, and maintained at − 80 °C to preserve RNA integrity for downstream qRT-PCR analysis. Gene expression dynamics were evaluated through RNA sequencing of potato samples exposed to 100 µM CdCl_2_·2.5H_2_O for 0 h, 24 h and 48 h. Each sample was replicated three times.

### RNA-seq data analysis

Total RNA was extracted and used to construct the RNA-seq library followed by high-throughput sequencing on an Illumina platform through Gene Denovo Biotechnology (Guangzhou, China). Raw sequencing data were deposited in the NCBI Sequence Read Archive (SRA) under accession PRJNA1020451.

Sequencing reads underwent rigorous quality filtering to generate high-confidence datasets. The cleaned data were validated by comparison with DM v6.1 gene models using HISAT2 software (v2.1.0). The data obtained from RNA sequencing were finally analyzed using TBtools software (v2.007).

### Differential expression genes analysis

Differential gene expression analysis was conducted using Fragments Per Kilobase of exon per Million fragments mapped (FPKM). Differentially expressed genes (DEGs) were defined by |FC| ≥ 2.0 and a *p*-value ≤ 0.05. Functional characterization of DEGs was achieved through comprehensive annotation against curated databases, including the non-redundant database (Nr), SwissProt/UniProt Plant Proteins, Kyoto Encyclopedia of Genes and Genomes (KEGG) and eggNOG. Enrichment analyses were conducted using KEGG and GO frameworks to identify significantly overrepresented biological processes and pathways.

### RNA isolation and qRT-PCR

Total RNA was purified using an RNA isolation kit (Tiangen DP452). cDNAs was synthesized using the Tiangen KR118 Kit and quantified by qRT-PCR with the Tiangen SYBR Green FP205 kit. Each sample was analyzed in triplicate. The 2^−ΔΔCt^ quantitative method was employed to determine the relative transcript levels of genes, with normalization against the elongation factor *ef1α* (GenBank: AB061263) as an endogenous control. Primer sequences for target amplification are provided in Supplementary Materials (Table S7). All results were visualized using TBtools.

### Statistical analysis

One-way ANOVA and Duncan’s multiple range tests were used for statistical significance of differences in gene expression. All experiments were analyzed using SPSS software (v22.0, SPSS, Inc., USA), at a statistically significant level (*P* < 0.05). All measurements were replicated three times.

## Supplementary Information

Additional file 1. Figure S1. Phylogenetic relationship, conserved motifs and gene structure of StCYP450 genes. A Phylogenetic relationship of StCYP450 genes. B The conserved motifs of StCYP450 genes were showed in different colors. C The exon-intron structure. The coding sequence (CDS) and untranslated region (UTR) were showed in different colors, and the lines between the boxes mean introns. Figure S2. The chromosomal mapping analysis of StCYP450 gene family in potato. Table S1. Physical and chemical properties of StCYP450 gene family in potato. AA. amino acid sequence length; MW. molecular weight; pI. isoelectric point; GRAVY. grand average of hydropathicity; II. instability index; AI. aliphatic index; SL. subcellular localization; CP. chloroplast; C. cytoplasm; CY. cytoskeleton; N. nucleus; V. vacuole; M. mitochondrion; PM. plasma membrane; E. extracellular matrix; ER. endoplasmic reticulum; P. peroxisome; G. golgi apparatus; C_N. cytoplasm_nucleus. Table S2. String protein in Arabidopsis corresponding to CYPs gene family in potato of phylogenetic tree. Table S3. Functional annotation of StCYP450 related to BRs and ABA in potatoes. Table S4. The GO classification of the annotated StCYP85 clan genes in potato. Table S5. FPKM values of StCYP72 and StCYP85 genes in various potato tissues. Table S6. FPKM values of StCYP72 and StCYP85 genes in potato different treatments. Table S7. Sequences of primer employed in the amplification of StCYP67 gene and qRT-PCR analysis.

## Data Availability

All data generated or analyzed during this study are included in this published article and its supplementary information files. The transcriptome data were deposited in the NCBI Sequence Read Archive (SRA) under accession PRJNA1020451.

## Abbreviations

BRs: Brassinosteroids
EBL: 24-epibrassinolide
NT: Non-transgenic plants
OE: StCYP67-overexpressed
Cd: Cadmium
