# Supplementary figures and images for: Comprehensive analysis and expression characterization of potato Cytochrome P450 gene family and the role of StCYP67 in abiotic stresses
Source: BMC Plant Biol. 2025 Sep 20;25:1207. doi: 10.1186/s12870-025-07392-y (PMC12449801; doi:10.1186/s12870-025-07392-y)

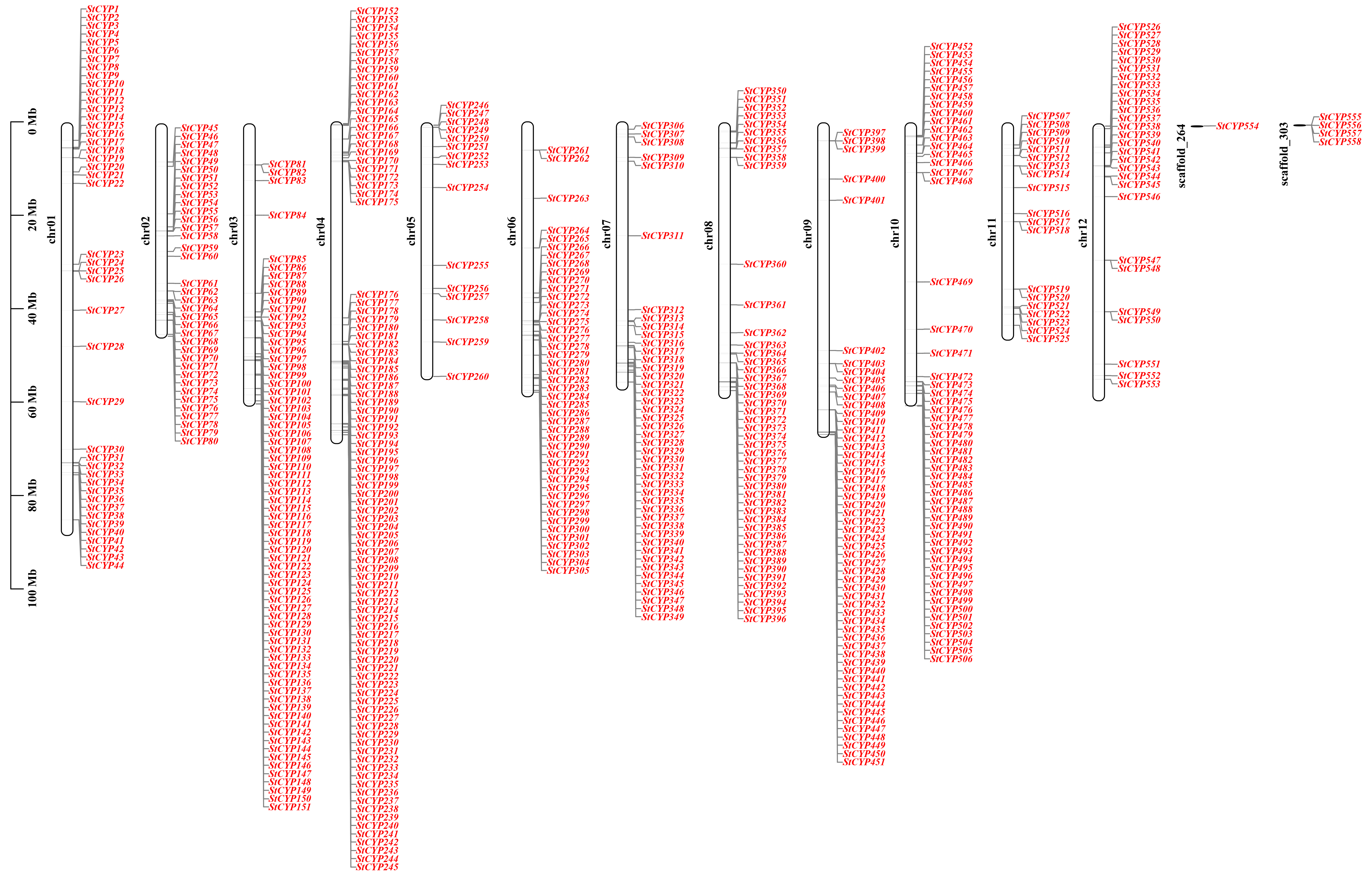

Supplement: Supplementary file 1 — Additional file 1. Figure S1. Phylogenetic relationship, conserved motifs and gene structure of StCYP450 genes. A Phylogenetic relationship of StCYP450 genes. B The conserved motifs of StCYP450 genes were showed in different colors. C The exon-intron structure. The coding sequence (CDS) and untranslated region (UTR) were showed in different colors, and the lines between the boxes mean introns. Figure S2. The chromosomal mapping analysis of StCYP450 gene family in potato. Table S1. Physical and chemical properties of StCYP450 gene family in potato. AA. amino acid sequence length; MW. molecular weight; pI. isoelectric point; GRAVY. grand average of hydropathicity; II. instability index; AI. aliphatic index; SL. subcellular localization; CP. chloroplast; C. cytoplasm; CY. cytoskeleton; N. nucleus; V. vacuole; M. mitochondrion; PM. plasma membrane; E. extracellular matrix; ER. endoplasmic reticulum; P. peroxisome; G. golgi apparatus; C_N. cytoplasm_nucleus. Table S2. String protein in Arabidopsis corresponding to CYPs gene family in potato of phylogenetic tree. Table S3. Functional annotation of StCYP450 related to BRs and ABA in potatoes. Table S4. The GO classification of the annotated StCYP85 clan genes in potato. Table S5. FPKM values of StCYP72 and StCYP85 genes in various potato tissues. Table S6. FPKM values of StCYP72 and StCYP85 genes in potato different treatments. Table S7. Sequences of primer employed in the amplification of StCYP67 gene and qRT-PCR analysis. [file 12870_2025_7392_MOESM1_ESM.zip › 12870_2025_7392_MOESM9_ESM.jpg]
